# Live Poultry Trade in Southern China Provinces and HPAIV H5N1 Infection in Humans and Poultry: The Role of Chinese New Year Festivities

**DOI:** 10.1371/journal.pone.0049712

**Published:** 2012-11-16

**Authors:** Ricardo J. Soares Magalhães, Xiaoyan Zhou, Beibei Jia, Fusheng Guo, Dirk U. Pfeiffer, Vincent Martin

**Affiliations:** 1 University of Queensland, School of Population Health, Infectious Disease Epidemiology Unit, Brisbane, Australia; 2 Food and Agriculture Organization of the United Nations, FAO HPAI H5N1 Programme in China, Beijing, China; 3 University of Prince Edward Island, Atlantic Veterinary College, Department of Health Management, Charlottetown, Canada; 4 University of London, The Royal Veterinary College, Department of Veterinary Clinical Sciences, Veterinary Epidemiology and Public Health Group, London, United Kingdom; University of Hong Kong, Hong Kong

## Abstract

**Background:**

The number of outbreaks of highly pathogenic avian influenza virus of the H5N1 subtype (HPAIV H5N1) over the past 5 years has been drastically reduced in China but sporadic infections in poultry and humans are still occurring. In this study, we aimed to investigate seasonal patterns in the association between the movement of live poultry originating from southern China and HPAIV H5N1 infection history in humans and poultry in China.

**Methodology/Principal Findings:**

During January to April 2010, longitudinal questionnaire surveys were carried out monthly in four wholesale live bird markets (LBMs) in Hunan and Guangxi provinces of South China. Using social network analysis, we found an increase in the number of observed links and degree centrality between LBMs and poultry sources in February and March compared to the months of January and April. The association of some live poultry traders (LPT’s) with a limited set of counties (within the catchment area of LBMs) in the months of February and March may support HPAIV H5N1 transmission and contribute to perpetuating HPAIV H5N1 virus circulation among certain groups of counties. The connectivity among counties experiencing human infection was significantly higher compared to counties without human infection for the months of January, March and April. Conversely, counties with poultry infections were found to be significantly less connected than counties without poultry infection for the month of February.

**Conclusions/Significance:**

Our results show that temporal variation in live poultry trade in Southern China around the Chinese New Year festivities is associated with higher HPAIV H5N1 infection risk in humans and poultry. This study has shown that capturing the dynamic nature of poultry trade networks in Southern China improves our ability to explain the spatiotemporal dissemination in avian influenza viruses in China.

## Introduction

China’s poultry sector plays an important role in the national economy [1]. The poultry sector is characterized by a traditional husbandry system (including backyard operations) which plays a key role in people’s livelihood and represents a significant part of the overall poultry output [1]. In addition, in the past decades there has been a rapid growth and concentration of large-scale commercial poultry production operations to meet strong and increasing consumer demand, concurrent with fast economic development in China.

Industrialization of livestock production is known to increase the risk of epidemics some of them with pandemic potential, as is the case with highly pathogenic avian influenza virus of the H5N1 subtype (HPAIV H5N1) [2]. HPAIV H5N1 poultry outbreaks in China have been reduced remarkably over the past five years particularly after the implementation of a control policy based on mass poultry vaccination. However, in the current epidemiological context whereby the clinical expression of the disease in poultry is becoming an exception, the silent circulation and likely persistence of influenza viruses, is an important challenge to disease control in China [3]–[7]. The resurgence of HPAIV H5N1 infection in early 2012 indicates that the risk for animal and human exposure to HPAIV H5N1 still persists in some segments of the poultry production and marketing industry in China [8], [9].

Available evidence indicates that live bird markets (LBMs) can serve as a possible mechanism by which infection is maintained for prolonged periods of time, posing additional risk for disease spread and human exposure [10]–[16]. In China, HPAIV H5N1 has regularly been detected in LBMs through the national surveillance program for the detection of HPAIV H5N1 circulation [6]. A recent study has shown that the genetic sequences of environmental (i.e. swabs of feces or bird droplets on the floors of cages and water on the floors or in ditches in the LBMs) and human isolates were highly similar, demonstrating a link between human H5N1 infection and the presence of the virus in LBMs [16]. This view is further supported by a case-control study conducted in Hong Kong where the exposure to live poultry at a LBM one week before illness was associated with a 4-fold increased risk in infection [17]. Another case-control study conducted in mainland China also showed that human urban cases were significantly more likely to have visited a LBM compared with rural cases [18].

To assist identifying persistence of infection or points of concentration along poultry market chains, methods initially developed in social sciences can be applied to epidemiology. Social network analysis (SNA) techniques provide a network based-approach and offer new insights on disease transmission dynamics, making it possible to develop more effective strategies for disease control [19], [20]. In the context of poultry market chains, SNA studies have been conducted in Asia to identify the core of a network using the topographical characteristics of the poultry trade network and quantify the risk associated with HPAIV H5N1infection along the market chain. For example, studies in Vietnam and Cambodia demonstrated the importance of evaluating live poultry movement and trading practices to develop appropriate and targeted surveillance recommendations for active HPAIV H5N1 surveillance programs [21], [22]. A recent study in Southern China has shown the relevance of this approach and provides a framework for analyzing the risk of HPAIV H5N1 along poultry marketing chains in the region [23]. More specifically, while providing new insights into the role of LBMs in China and HPAIV H5N1 presence, that study demonstrated that network parameters – such as degree centrality and *k*-core – are highly relevant for better understanding infection risk. However, these studies were based on the analysis of static networks and have not evaluated network connectivity comparing consecutive periods in time. This knowledge would improve our ability to identify temporal features in risk presenting opportunities to temporal targeting of interventions. Additionally, as opposed to the cross-sectional approach adopted in most previous studies (in which network characteristics where based on a single assessment), a longitudinal approach for studying LBM networks (in which network characteristics are sequentially evaluated at multiple time points) would better capture the dynamic nature of these networks which are particularly sensitive to seasonal variation.

In this study, a comprehensive longitudinal LBM survey was implemented in January to April 2010 in South China in Hunan province and Guangxi autonomous region, combining outbreak reporting, virus surveillance surveys and social network analysis. We aimed to investigate associations between poultry trade network characteristics in southern China and HPAIV H5N1 infection status represented by the historical occurrence of poultry outbreaks, human cases or HPAIV H5N1 isolated in LBMs.

## Methods

### Ethics Statement

The research proposal leading to the study received official approval from the Veterinary Bureau of the Ministry of Agriculture of the People’s Republic of China (MoA). Ethical approval for the questionnaire survey at the Yangjiashan, Shima and Wuliting wholesale markets and Wuyizhonglu waterfowl market was obtained from the ethics committees of the provincial and prefectural official animal health agencies in Hunan province and Guangxi autonomous region, respectively. Participation in the questionnaire survey was voluntary and questionnaire data collection procedures were only conducted after verbal consent had been obtained from participants. To ensure participant confidentiality individual questionnaire records were anonymized using coded labels and all data analyses were carried out in anonymized data entries.

### Data Sources

Monthly questionnaire-based surveys were carried out during January to April 2010 in two wholesale LBMs in Hunan province (Yangjiashan wholesale market and Shima wholesale market) and two LBMs in Guangxi autonomous region (Wuliting wholesale market and Wuyizhonglu waterfowl market). These LBMs had been included as part of a larger cross-sectional network study that included a total of 30 LBMs in the provinces of Hunan, Yunnan and Guangxi autonomous region [23]. For the purpose of our monthly assessment we selected the Hunan province and Guangxi autonomous region purposively primarily because HPAIV H5N1 had occurred in the past and the existence of a traditional poultry production and marketing systems which are considered to play a significant role in HPAIV H5N1 epidemiology. Within these provinces, the four LBMs were also selected purposively on the basis of information on size and volume of poultry marketing which had previously been provided by animal health officials at the Animal Disease Prevention and Control Center in the provincial capital cities (i.e. Nanning and Changsha) [23]. In brief, the Wulitin market is the only whole-sale market for the provincial capital Nanning and the Yangjiashan and Shima markets were the only two whole-sale markets in the provincial capital Changcha. While there are many other LBMs in the provinces where we targeted our study, the four markets included in the study represent the major trading situation of all the LBMs in the two capital cities. The time period before and after the Chinese New Year public holiday period (February 14, 2010) was selected to represent a key period with change in poultry consumption demand and therefore production quantity and trade intensity are known to occur.

Recruitment of live poultry traders (LPTs) and data collection procedures for the construction of the poultry networks has been described previously [23]. In brief, each visit to the LBMs was conducted in the morning at a time when all stalls were occupied by LPTs and poultry entry records were consulted from the market manager to identify all large-scale LPTs available during that morning which were subsequently invited to participate – this lead to the recruitment of LPTs which had at the moment of the survey 80% of the volume of poultry. While on average the markets had 80–90 traders and different LPTs were interviewed during each visit, all LPTs trading large volumes of live poultry (responsible for the majority of trade fluctuation of that market) were interviewed in each visit. Data collection for the construction of the poultry networks has followed an ego-centric approach whereby poultry trading activities were ascertained by the personal account of live poultry traders (LPTs) present at the markets using a standardized questionnaire and further links mentioned by LPTs were not followed up. In the questionnaire, LPTs were asked about (1) their poultry trade activities outside and inside the LBM and (2) their relationship with other intermediaries and poultry flocks. The questionnaire included specific questions regarding the location at the county level of the flocks from which they had collected their poultry and to the number of poultry involved in the movement. The latter contained many missing values and was not considered in further analyses.

HPAIV H5N1 infection data in poultry and humans were collected following the procedures detailed elsewhere [23]. In brief, poultry HPAIV H5N1 outbreak data from early 2004 to October 2010 were compiled from the Official Veterinary bulletin published on the MoA website (http://english.agri.gov.cn/). Data on HPAIV H5N1 human cases covering the same temporal period were obtained from the Ministry of Health of the People’s Republic of China website (http://www.moh.gov.cn/) as well as the World Health Organization website (http://www.who.int/csr/don/en/) and geo-coded according to the geographical source of infection. Data on the presence of HPAIV H5N1 in LBMs was obtained from previous virological surveys carried out in Southern China [23] and from the monthly provincial LBM surveillance program coordinated by MoA. These data were combined since the surveillance protocols and laboratory testing methods in both surveys were similar. The Yangjiashang and Shima wholesale markets are located in counties with previous a history of HPAIV H5N1 infection in poultry and humans. The Wulitin and the Wuyizhonglu markets are located in counties with previous history of HPAIV H5N1 infection in poultry but not in humans. Poultry within all markets had been diagnosed with HPAIV H5N1 serosurveillance.

The administrative level “county” was used to define infection status of the location of trading events. Data on HPAIV H5N1 presence in markets, poultry outbreak and human cases at county level, hereafter referred as HPAIV H5N1 infection status were used as dependent variables in our study. Thereby, for each county in the network database its infection status was linked to its geographic centroid location in a geographic information system, ArcGIS 10 (^©^ ESRI).

### Social Network Analysis

Social network analysis (SNA) was used to describe the connectivity pattern within the network dataset consisting of records of paired trading events [24]. Each pair represented the binary link between a particular LBM and the county of origin of the purchased poultry. For the purpose of the paper from hereafter the county of origin of purchased poultry will be termed as “source”. The networks are termed binary because the links between network nodes (i.e. LBM or source) are defined as whether a node is linked (taking a value of “1”) or not (taking a value of “0”) to another node. We summarised network connectivity of all networks using the number of links, degree centrality (number of unique links), *k*-core (sub-group within a network in which each node has at least *K* links between each other) and the components of the network [a maximal connected subgraph where all nodes (i.e. poultry sources) are connected through paths].

We built for each survey month, one symmetric 2-mode binary networks (Network 1; LBM-source network), linking LBMs and sources. This network is named 2-mode because nodes are divided into two classes: LBMs and sources. In Network 1, two sources are linked via a common LBM if the LPTs (in that LBM) reported to have bought poultry from flocks in both sources during the study period.

Although centrality measures at node level (such as the degree and membership of the giant component) have been suggested to be of practical use in the development of effective targeted disease control strategies, the investigation of the links within and between subgroups of nodes has provided better insight than analysis of degree distribution and component membership into the relationship between the disease status and network structure [21]. To investigate differences in network parameters with respect to type of links established within the subgroup of sources, the 2-mode LBM-source network was converted into one 1-mode binary symmetric network of sources (Network 2; source-source network). The links established between poultry sources were classified based on the different variables of infection status: Type 1 link – between infected sources, Type 2 link– between infected and non-infected sources, and Type 3 link – between non-infected sources.We did not construct the 1-mode LBM-LBM network nor carry out the same sort of analysis because we only had 4 LBMs.

### Statistical Analysis

To evaluate whether the degree centrality and *k*-core of both LBMs and sources varied with time we used network estimates from 2-mode networks (Network 1) for each month. Statistical associations were tested using generalised estimating equations models (GEEs). To take into account the overdispersed nature of the degree and *k*-core data we parameterised the GEE models with a negative binomial family function and a log link function. In these models degree centrality and *k*-core were the dependent variables and the month of survey were the independent variable and the source ID the unit of analysis. These analyses were carried out in Stata 11 (Stata® Corp.).

Several statistical tests adapted to network data were applied to the 1-mode source-source network (Network 2) to test the association between degree centrality of poultry sources and HPAIV H5N1 infection status of the poultry source for each month [25]. First, the means of degree centrality of infected and non-infected counties were compared using a *t*-test with a permutation-based significance test involving 10,000 random permutations. The association between the density of the links in Network 2 and infection status of the source was tested by applying a randomization test of autocorrelation for a symmetric adjacency matrix using two classes and 10,000 random permutations. Second, we compared the observed number of links between two groups of nodes to the expected number obtained through random permutation of Network 2, using a test of autocorrelation. The use of randomization tests of autocorrelation within symmetric adjacency matrices allows statistical significance testing of associations between dyadic binary variables such as represented by the links within the network and the infection status attributes of the counties [24], [26]. All SNAs were performed using UCINET 6.135 (^©^Analytic Technologies, Inc. 1999). Maps of the study area and the centroid coordinate location of the communes included in the networks were produced using ArcGIS 10 (^©^ ESRI).

## Results

During January and April 2010, a total of 25 LPTs were interviewed monthly in each LBM, resulting in a total record of 513 trading events. Most trading events were recorded in the markets selected in Hunan province, particularly Yangjiashang whole sale market (n = 176; 34%) followed by Shima wholesale market (n = 142; 28%). The Wulitin and the Wuyizhonglu markets in Guangxi province represented 24% (n = 125) and 14% (n = 70) of the trading events recorded, respectively. Regarding the 182 poultry sources identified in the questionnaire, 25 (14%) were located in counties with previous history of human infection, 42 (23%) with previous history of poultry infection and 47 (26%) in countries with previous history of poultry serosurveillance positive to HPAIV H5N1. We were unable to ascertain if human or poultry outbreaks were epidemiologically linked.

### LBM-source Networks

The January Network 1 comprised a total of 109 links between study LBMs (n = 4) and poultry sources (n = 66) with an average degree of 2.1 (range: 1–27) and an average *K*-core of 1.1 (range: 1–2). The February Network 1 comprised a total of 122 links between study LBMs (n = 4) and poultry sources (n = 85) with an average degree of 2.1 (range: 1–33) and an average *K*-core of 1.1(range: 1–2). The March Network 1 comprised a total of 121 links between study LBMs (n = 4) and poultry sources (n = 77) with an average degree of 2.2 (range: 1–34) and an average *k*-core of 1.2 (range: 1–2). The April Network 1 comprised a total of 79 links between study LBMs (n = 4) and poultry sources (n = 60) with an average degree of 2.1 (range: 1–27) and an average *k*-core of 1.1 (range: 1–2). While the networks for February and March had 1 component each, there were two components for each of the months of January (27 and 43 nodes) and April (26 and 38 nodes).

**Figure 1:**
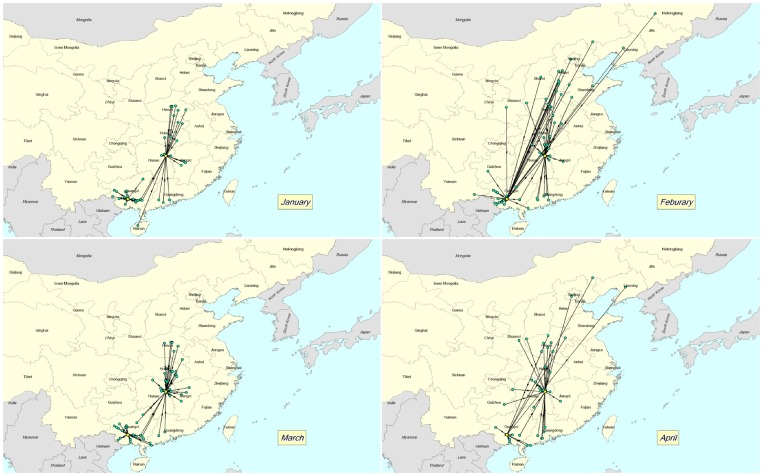
Geographical distribution of counties involved in live bird market networks originating from southern China in the (A) January, (B) February, (C) March and (D) April.

In addition, we found a difference in the geographical extent of the network between the months of January and April, with the maximum distance between counties occurring in February (Figure 1). The average geographical distances were in January 356 km (range: 13–1,222), in February 803 km (range: 2–3,709), in March 344 km (range: 2–1,356) and in April 592 km (range: 2–3,730).

The mean degree centrality for the months of February and March is significantly higher than that of January (P<0.001); in contrast, the mean degree centrality for April is lower compared to the month of January (*P* = 0.047) (Table 1). Our results also show that the mean *K*-core was not significantly different between the months of January to April (Table 1).

**Table 1:** Results of analysis of associations between network parameters (degree centrality and *k*-core) and month of survey, based on parameters extracted from Network 1 and using generalised estimating equation models.

	Degree centrality		*k*-core	
Month of survey	Coefficient (95% CI)	P>z	Coefficient (95% CI)	P>z
*February (vs January)*	0.22 (0.12, 0.31)	<0.001	0.16 (−0.17, 0.50)	0.345
*March (vs January)*	0.20 (0.10, 0.29)	<0.001	0.14 (−0.20, 0.48)	0.414
*April (vs January)*	−0.10 (−0.20, −0.002)	0.047	−0.12 (−0.47, 0.23)	0.505
*Intercept*	−0.15 (−0.37, 0.08)	0.195	−0.75 (−1.01, −0.48)	<0.001

The mean degree centrality of the LBMs during January to April is significantly increased in the months of February [23.0; standard deviation (SD): 9.20] and March (22.5; SD: 9.85) compared to January (18.50; SD: 6.61) and April (16.50; SD: 5.80).

### Source-Source Networks

The symmetric binary 1-mode source network (Network 2) included 68 nodes establishing 1,412 links in January, 85 nodes establishing 2,258 links in February, 77 nodes establishing 2,136 links in March and 60 nodes establishing 1,104 links in April. The average degree was in January 20.8 (range: 10–41), in February 26.6 (range: 10–42), in March 27.4 (range: 9–68) and in April 18.4 (range: 9–35). The average *k*-core in January was 19.6 (range: 10–26), in February was 25.3 (range: 10–32), in March was 25.2 (range: 9–33) and in April was 17.2 (range: 9–23). While the networks for February and March had 1 component each, there were two components for each of the months of January (26 and 42 nodes) and April (24 and 36 nodes).

Applying significance tests to the data on source HPAIV H5N1 infection status (poultry/human outbreak and market surveillance data), we found that the connectivity of counties that had human infection was consistently higher between January and April compared to counties with no human infection; this was statistically significant in January (P = 0.001), March (P = 0.011) and April (P = 0.018) (Table 2). In contrast, the connectivity of counties that had poultry infection was lower compared to counties with no poultry infection detected; this was only statistically significant in February (P = 0.018).

**Table 2:** Comparison of mean degree between non-infected and infected poultry sources (Network 2) for different source infection status categories, during January to April.

Month/County infection status	Not-infected	Infected	Two-tailed t-test probability of the difference of the mean degree
**January**			
*Poultry outbreaks*	47	21	−0.203 (P = 0.933)
*Market infection*	43	25	0.577 (P = 0.768)
*Human outbreaks*	53	15	−6.974 (P = 0.001)
**February**			
*Poultry outbreaks*	61	24	4.561 (P = 0.018)
*Market infection*	59	26	5.475 (P = 0.001)
*Human outbreaks*	68	17	−1.941 (P = 0.202)
**March**			
*Poultry outbreaks*	57	20	5.325 (P = 0.076)
*Market infection*	57	20	5.325 (P = 0.078)
*Human outbreaks*	65	12	−8.559 (P = 0.011)
**April**			
*Poultry outbreaks*	41	19	1.972 (P = 0.324)
*Market infection*	40	20	2.100 (P = 0.281)
*Human outbreaks*	49	11	−5.187 (P = 0.018)

The test of autocorrelation for Network 2 for each month showed that the observed number of links between counties reporting poultry infection at markets and poultry outbreaks was significantly higher than expected under randomness from February to April (Table 3). Yet the observed number of links between counties reporting human infection was marginally higher than expected in February (P = 0.054) and significantly higher than expected in March (P = 0.031). The proportion of links between infected and non-infected counties (Type 2) was significantly lower than expected for all infection status variables (P<0.001) for the months of February and March.

**Table 3:** Ratio between observed and expected Type 1, 2 and 3 links (with two-tailed *t*-test *p*-value) for different source infection status categories, during January to April.

Month/Source infection status	Not-infected	Infected	Type 1 links	Type 2 links	Type 3 links	*p*-value
**January**						
*Poultry outbreaks*	47	21	1.23	0.66	1.26	0.799
*Market infection*	43	25	1.20	0.67	1.32	0.731
*Human outbreaks*	53	15	2.39	0.67	1.09	0.081
**February**						
*Poultry outbreaks*	61	24	1.28	0.48	1.37	0.001
*Market infection*	59	26	1.39	0.42	1.45	<0.001
*Human outbreaks*	68	17	1.84	0.56	1.18	0.054
**March**						
*Poultry outbreaks*	57	20	1.43	0.75	1.35	0.001
*Market infection*	57	20	1.52	0.67	1.44	<0.001
*Human outbreaks*	65	12	1.97	0.42	1.73	0.031
**April**						
*Poultry outbreaks*	41	19	1.54	0.47	1.40	0.004
*Market infection*	40	20	1.46	0.48	1.42	0.004
*Human outbreaks*	49	11	2.44	0.64	1.10	0.127

Type 1 link – between infected sources; Type 2 link - between infected and non-infected sources; Type 3 links – between non-infected sources.

## Discussion

This study provides important new knowledge with regard to the temporal dynamics of live poultry trade during the months around the Chinese New Year in counties of South China and how this information is associated with retrospective data on HPAIV H5N1 infection reported in poultry and humans in China. The findings of this study extend previous SNA studies by specifically investigating the temporal variation in network topology. In this respect our findings provide new insights that will allow the development of HPAIV H5N1 control strategies along poultry marketing chains adapted to temporal changes in risk.

Despite the connectivity (as measured by *k*-core membership) between LBM and poultry sources (Network 1) remaining stable during the study period, in February and March there was an increase by about 30% in the number of links and degree centrality compared to the months of January and April. An LBM that has a high degree centrality makes contacts with more poultry sources. Therefore, the existence of temporal variation in the degree centrality of LBMs suggests a greater opportunity for infection to propagate during the months of high poultry demand should HPAIV H5N1-infected poultry flow through the marketing channel. This finding has important implications in the context of disease control at the level of LBMs because the degree centrality of network nodes infected early in an outbreak may determine whether of not an epidemic emerges [27]. More importantly, we also found that the geographical extent of poultry trade is greater during February compared to other months surveyed indicating that the marketing chain of poultry products originating in south China can reach wider geographical coverage during this month. This result is in contrast with a network study in Cambodia which noted that while the volume of poultry being traded drastically increased in the weeks prior to the Chinese and Khmer New Years festivals, the locations where poultry were traded remained the same [22]. In addition, we also found that in the months of January and April poultry sources and LBMs share their network membership through two separate components; however, in February and March all nodes belong to the same component. The presence of a highly connected core is likely to pose considerable challenges for the containment of HPAIV H5N1 provided infected poultry flow through the marketing channel. Taken together, these results highlight the role of live bird markets in South China in facilitating the seasonal movement of live poultry between southern and northern provinces of China, probably due to the high demand during and shortly after the Chinese New Year festivities.

Recent molecular and epidemiological investigations indicate a role of poultry trade on the large scale dissemination of HPAIV H5N1 clades [16], [28]. Network parameters such as *k*-core and degree centrality can be used to assess the potential impact of each network node as diffusers in the network. The *k*-core can reflect the spread of an infection through a poultry network provided that any poultry moved from an infected source results in the infection of all linked nodes with a maximum *k* distance and that all poultry moved to other sources are infectious. The membership of poultry sources (Network 2) to sub-groups of higher *k*-core during the months of February and March compared to January and April suggests an increased potential for wider infection diffusion during the months of Chinese New Year. Analysis of same network also shows an increase in the degree centrality of poultry sources during the months of February and March (compared to January and April) associated with increased risk of HPAIV H5N1 infection. With that regard, we found that the association between the degree centrality of poultry sources and the circulation of HPAIV H5N1 in poultry (reported outbreaks and market surveillance positive results) and in humans (reported outbreaks) varies with time. The results indicate that the degree centrality of counties that had poultry outbreaks (or positive market surveillance) is lower compared to counties with no poultry infection detected. This finding is statistically significant in February and marginally significant in March suggesting that, during peak poultry trade, areas with previous history of HPAIV H5N1 appear to be less involved in poultry trade compared to areas without historical records of HPAIV H5N1 infection presence suggesting that LPTs in peak trading time may take into account previous history of HPAIV H5N1 and avoid areas where poultry outbreaks had occurred. In contrast, our results for human outbreaks indicate that the connectivity of counties which reported human HPAIV H5N1 infection is significantly higher during most of the study period compared to counties with no human infection consolidating the view that marketing of poultry is a risk factor for the transmission of HPAIV H5N1 infection to humans. The observed association between network degree centrality and human infection (reported H5N1 cases in humans) suggests that the association of some LPTs with a limited set of counties (within the catchment area of LBMs) during the peak poultry trade period may support HPAIV H5N1 transmission and may contribute to perpetuating HPAIV H5N1 virus circulation among some groups of counties in China.

Using the network of poultry sources (Network 2), we analysed the role played by the source of poultry in supporting HPAIV H5N1 presence in LBMs and possibly its persistence within certain poultry network configurations. Overall our findings corroborate previous network studies which postulated limited spread of infection within the identified network, under the assumption that HPAIV H5N1 transmission was only to occur through poultry trade relations between these counties [21], [23]. This is demonstrated by the higher number of links between counties or source nodes having the same status (i.e. infected-infected or free-free) while the number of links between free and infected counties is generally significantly lower than expected. This association was consistently found for the three categories of infection statuses investigated. Furthermore, and applying statistical significance tests, our results provide for the first time evidence of temporal variation in the number of links between infected counties. These analyses highlight that poultry movement between counties with human infection appears to be significantly increased shortly after the annual festivities of Chinese New Year (i.e. during the month of March). In contrast, our results for poultry infection (reported outbreaks and positive LBM surveillance) suggest that poultry movement significantly associated with infection in poultry is initiated during those festivities, peaking in March and losing significance in April. These findings are consistent with the temporal pattern of recent poultry and human outbreaks in China in that poultry outbreak reports have preceded human cases [8], [9]. Thus, these results represent important new knowledge showing that there is temporal variation in HPAIV H5N1 infection risk associated with poultry trade and that this risk differs between poultry and human hosts.

A number of study limitations should be noted. Firstly, although we have targeted our longitudinal surveys to a 4-month period to maximize the chance of detecting seasonal patterns in poultry movement, in doing so we may have missed other periods of the year which may also be associated with the observed infection in poultry and humans. Secondly, we targeted our surveys to two large wholesale markets in two provinces of southern China which are considered to have a significant role in poultry trade in China. We acknowledge that there may be other LBMs which may also play an important role in explaining the observed pattern of HPAIV H5N1 infection in China. Thirdly, although we aimed to capture all relational information from LPTs there is also the risk that some movements may have been missed and therefore the networks may not represent all poultry movements. The ego-centric approach studies the networks of relations surrounding individuals rather than focusing on the complete network linking all individuals. Finally, we used retrospective HPAIV H5N1 data which was aggregated at county level and thus it constitutes an imperfect measure of exposure. Despite these limitations it is noteworthy that we have identified a strong signal in our network data indicating that live poultry trade is significantly associated with human and poultry HPAIV H5N1 outbreaks during the peak movement season in China. Future studies are needed to address whether geographical differences between H5N1-infected and non-infected areas can account for the differences in network parameters identified in this study.

We have demonstrated that a longitudinal approach for studying LBM networks improves our understanding of the seasonal effects of poultry movement on HPAIV H5N1 infection in humans and poultry within the catchment of LBMs. Our approach generated detailed network information that improves our ability to explain the spatiotemporal dissemination of avian influenza viruses in China. In addition, by capturing the dynamic nature of these networks in south China, it also allowed the quantification of temporal variation in the geographical extent of live poultry movements originating in LBMs in southern China within and beyond the region. Given the above, current disease prevention and control interventions would benefit from an increased knowledge about poultry trading patterns based on a continuous market-based formal data recording system.

## References

[1] Bingsheng K, Han Y (2008) Poultry Sector in China: Structural Changes in the Past Decade and Future Trend. Rome.

[2] Leibler JH, Otte J, Roland-Holst D, Pfeiffer DU, Soares Magalhaes R, et al (2009) Industrial food animal production and global health risks: exploring the ecosystems and economics of avian influenza. Ecohealth 6: 58–70. 19437076 10.1007/s10393-009-0226-0 PMC7087879

[3] Gilbert M, Chaitaweesub P, Parakamawongsa T, Premashthira S, Tiensin T, et al (2006) Free-grazing ducks and highly pathogenic avian influenza, Thailand. Emerg Infect Dis 12: 227–234. 16494747 10.3201/eid1202.050640 PMC3373083

[4] Gilbert M, Xiao X, Pfeiffer DU, Epprecht M, Boles S, et al (2008) Mapping H5N1 highly pathogenic avian influenza risk in Southeast Asia. Proc Natl Acad Sci U S A 105: 4769–4774. 18362346 10.1073/pnas.0710581105 PMC2290786

[5] Pfeiffer DU, Minh PQ, Martin V, Epprecht M, Otte MJ (2007) An analysis of the spatial and temporal patterns of highly pathogenic avian influenza occurrence in Vietnam using national surveillance data. Vet J 174: 302–309. 17604193 10.1016/j.tvjl.2007.05.010

[6] Martin V, Pfeiffer DU, Zhou X, Xiao X, Prosser DJ, et al (2011) Spatial distribution and risk factors of highly pathogenic avian influenza (HPAI) H5N1 in China. PLoS Pathog 7: e1001308. 21408202 10.1371/journal.ppat.1001308 PMC3048366

[7] Shortridge KF, Peiris JS, Guan Y (2003) The next influenza pandemic: lessons from Hong Kong. J Appl Microbiol 94 Suppl: 70S–79S 12675938 10.1046/j.1365-2672.94.s1.8.x

[8] OIE (2011) Highly pathogenic avian influenza, China.

[9] WHO (2012) Situation updates - Avian influenza.

[10] Nguyen DC, Uyeki TM, Jadhao S, Maines T, Shaw M, et al (2005) Isolation and characterization of avian influenza viruses, including highly pathogenic H5N1, from poultry in live bird markets in Hanoi, Vietnam, in 2001. J Virol 79: 4201–4212. 15767421 10.1128/JVI.79.7.4201-4212.2005 PMC1061558

[11] Choi YK, Seo SH, Kim JA, Webby RJ, Webster RG (2005) Avian influenza viruses in Korean live poultry markets and their pathogenic potential. Virology 332: 529–537. 15680418 10.1016/j.virol.2004.12.002

[12] Wang M, Di B, Zhou DH, Zheng BJ, Jing H, et al (2006) Food markets with live birds as source of avian influenza. Emerg Infect Dis 12: 1773–1775. 17283635 10.3201/eid1211.060675 PMC3372357

[13] Bulaga LL, Garber L, Senne DA, Myers TJ, Good R, et al (2003) Epidemiologic and surveillance studies on avian influenza in live-bird markets in New York and New Jersey, 2001. Avian Dis 47: 996–1001. 14575100 10.1637/0005-2086-47.s3.996

[14] Panigrahy B, Senne DA, Pedersen JC (2002) Avian influenza virus subtypes inside and outside the live bird markets, 1993–2000: a spatial and temporal relationship. Avian Dis 46: 298–307. 12061638 10.1637/0005-2086(2002)046[0298:AIVSIA]2.0.CO;2

[15] Liu M, He S, Walker D, Zhou N, Perez DR, et al (2003) The influenza virus gene pool in a poultry market in South central china. Virology 305: 267–275. 12573572 10.1006/viro.2002.1762

[16] Wan XF, Dong L, Lan Y, Long LP, Xu C, et al (2011) Indications that live poultry markets are a major source of human H5N1 influenza virus infection in China. J Virol 85: 13432–13438. 21976646 10.1128/JVI.05266-11 PMC3233185

[17] Mounts AW, Kwong H, Izurieta HS, Ho Y, Au T, et al (1999) Case-control study of risk factors for avian influenza A (H5N1) disease, Hong Kong, 1997. J Infect Dis 180: 505–508. 10395870 10.1086/314903

[18] Zhou L, Liao Q, Dong L, Huai Y, Bai T, et al (2009) Risk factors for human illness with avian influenza A (H5N1) virus infection in China. J Infect Dis 199: 1726–1734. 19416076 10.1086/599206 PMC2759027

[19] Klovdahl AS, Graviss EA, Yaganehdoost A, Ross MW, Wanger A, et al (2001) Networks and tuberculosis: an undetected community outbreak involving public places. Soc Sci Med 52: 681–694. 11218173 10.1016/s0277-9536(00)00170-2

[20] Rothenberg RB, Potterat JJ, Woodhouse DE, Muth SQ, Darrow WW, et al (1998) Social network dynamics and HIV transmission. Aids 12: 1529–1536. 9727575 10.1097/00002030-199812000-00016

[21] Soares Magalhaes RJ, Ortiz-Pelaez A, Lai Thi KL, Hoang DQ, Otte J, et al. (2010) Associations between attributes of live poultry trade and HPAI H5N1 outbreaks: a descriptive and network analysis study in northern Vietnam. BMC Veterinary Research 6: doi:10.1186/1746-6148-1186-1110. 10.1186/1746-6148-6-10 PMC2837645 20175881

[22] Van Kerkhove MD, Vong S, Guitian J, Holl D, Mangtani P, et al (2009) Poultry movement networks in Cambodia: implications for surveillance and control of highly pathogenic avian influenza (HPAI/H5N1). Vaccine 27: 6345–6352. 19840671 10.1016/j.vaccine.2009.05.004

[23] Martin V, Zhou X, Marshall E, Jia B, Fusheng G, et al (2011) Risk-based surveillance for avian influenza control along poultry market chains in South China: The value of social network analysis. Prev Vet Med 102: 196–205. 21925753 10.1016/j.prevetmed.2011.07.007 PMC7127115

[24] Scott J (2000) Social Network Analysis: A Handbook. London: SAGE Publications Ltd.

[25] Wasserman S, Faust K (1994) Social Network Analysis: Methods and Applications. Cambridge: Cambridge University Press.

[26] Hanneman R, Riddle M (2005) Introduction to Social Networks.

[27] Christley RM, Pinchbeck GL, Bowers RG, Clancy D, French NP, et al (2005) Infection in social networks: using network analysis to identify high-risk individuals. American journal of epidemiology 162: 1024–1031. 16177140 10.1093/aje/kwi308

[28] Pfeiffer DU, Otte MJ, Roland-Holst D, Inui K, Nguyen T, et al (2011) Implications of global and regional patterns of highly pathogenic avian influenza virus H5N1 clades for risk management. Vet J 190: 309–316. 21288747 10.1016/j.tvjl.2010.12.022

